# Learning environment at home and primary school dropout status in Bangladesh: An analysis of multiple indicator cluster survey

**DOI:** 10.1371/journal.pone.0360931

**Published:** 2026-10-21

**Authors:** Md. Emon Prodhan, Ashraful Alam, Sajida Subha, Faizul Islam, Mohd. Al Amin, Mohammad Nayeem Hasan, Mohammad Anamul Haque, Md Jamal Uddin

**Affiliations:** 1 Department of Statistics, Shahjalal University of Science & Technology, Sylhet, Bangladesh; 2 Department of Computer Science & Engineering, Daffodil International University, Savar, Dhaka, Bangladesh; Akademia Jagiellońska w Toruniu: Akademia Jagiellonska, CZECHIA

## Abstract

Primary school dropout is a serious concern worldwide. In Bangladesh, primary education is strongly shaped by the home learning environment. This study aimed to investigate the associations between the dropout of primary school children and the learning environment at home as well as the associated risk factors by analyzing cross-sectional data from the 2019 Multiple Indicator Cluster Survey (MICS), with a total sample size of 14,278 children aged 6–12 years. Bivariate analysis, crude and adjusted log-binomial models were used to assess the relationships between dropout and risk factors. Overall, 1.68% of the children dropped out, with increased rates observed among males (ARR = 2.44, 95% CI: 1.63–3.64) and children aged 9–12 years at the beginning of the school year had a higher risk of dropout (ARR = 1.83, 95% CI: 1.15–2.93). Mymensingh presented the highest dropout rate, 3.82 (ARR = 3.82, 95% CI: 1.59–9.18) times higher than Barishal. Children who did not read books or were not read to at home had 14.47 (ARR = 14.47, 95% CI: 9.30–22.51) times higher risk of primary school dropout. To reduce primary school dropouts in Bangladesh, addressing gender inequalities, regional disparities, and inadequate home reading activity is essential. Strengthening early childhood education initiatives, promoting home-based literacy activities, supporting older children in their grades, and implementing targeted interventions in disadvantaged regions may help reduce primary school dropout in Bangladesh.

## Introduction

Education is key to economic growth, employment, poverty reduction, economic independence, and self-awareness of happiness [1–3]. Primary education has a substantial positive impact on economic development, allowing citizens to live stable lives and supporting sustainable living [4,5]. The learning environment is the basis of a child’s learning process and decisions regarding their future workplaces. It is considered a crucial element in determining the effectiveness of an efficient curriculum and the academic achievements of students [6]. Therefore, it is necessary to understand why people decide to drop out of school in developing countries, particularly in low- and middle-income countries [7,8]**.** A dropout is a student who has left school before completing the prescribed curriculum and has not enrolled in the same or different schools during the following academic session [9]. Dropping out of school is a major issue that has an impact on individuals, the community, the educational system, and society at large.

The primary school dropout for millions of children continues to be a major concern in low- and middle-income nations. Sub-Saharan Africa has the highest dropout rate (42%), followed by South Asia and West Asia (33%) [10]. The evidence that there are an estimated 20.6 million lower secondary dropout children and 11.3 million elementary school-age dropout children in South Asia is supported by data gathered by the United Nations Development Programme (UNDP) [11]. An investigation revealed that households with better learning environments are associated with higher child academic outcomes. Children who have supportive parents perform better academically and behaviorally [12]. Economic status and interrelated social, school, and cultural factors are the root causes of dropout [13]. The income and educational levels of parents have detrimental effects on dropouts [14]. Factors such as gender, religion, ethnicity, housework, wage labor, disability, low self-esteem among children, poverty, and profanity in education among children are recognized as major causes of school dropout, with additional correlates such as late entry, grade repetition, mediocre educational objectives, and a 9% increase in the likelihood of dropout linked to the loss of the family head’s job [15–17]. There is a significant relationship among age, race, corporal punishment, and school dropouts, which shows that dropping out of school contributes not only to a cycle of violent victimization but also to educational disparities, long-term unemployment, poverty, poor health outcomes, continued reliance on public assistance, political and social apathy, and juvenile crime [18–23]. UNESCO has reported that continuing school dropout and educational gaps impose substantial economic costs globally, with losses projected to reach US$10 trillion annually by 2030, disproportionately affecting low- and middle-income countries [24].

In Bangladesh, the primary school dropout rate was 14.15% in 2021 [25]. Additionally, evidence suggests that the proportion of rich children who complete school is 22 percent greater than that of poor children [26]. Households in the richer and richest wealth groups have lower school dropouts, whereas children living in a larger family or having siblings have higher school dropouts [27]. The risk that a daughter will drop out of school increases as fathers participate more in home decision-making [28]. Age, gender, marriage, and financial constraints are the top predictors of dropout in Bangladesh. Dropout continues to be strongly correlated with parental disengagement from their children’s academics and absences from school [29–31]. With respect to low-income families, the children (41%) had to take care of their homes, which included helping parents with household tasks (cooking or agricultural work) or raising a sibling or younger child [32]. However, a further study explored the factors contributing to school dropout in Bangladesh, including persistent poverty, parents’ unwillingness, financial obstacles, biased social practices, poor school infrastructure, lack of quality education, geographic isolation, unequal access to education, and security issues for girls [33]. Although several studies have been conducted on dropout, no study has examined the association between primary school dropout and the home learning environment in Bangladesh. This study aims to investigate the association between home learning environment and primary school dropout in Bangladesh. This study uniquely examines household-level stimulation conditions, assessing whether children growing up in environments that encourage reading are more likely to remain enrolled in school. The findings can provide policymakers and development stakeholders who wish to reduce dropout rates in low- and middle-income countries. Household-targeted interventions, such as initiatives for educating parents, low-cost learning materials, and community-led tutoring support, may help children from poor families overcome barriers to learning. National education monitoring systems may enhance their effectiveness by incorporating home learning environment indicators, especially in relation to Sustainable Development Goal 4, which aims for inclusive and equitable quality education.

## Methods

### Data source

We used the latest dataset of the Multiple Indicator Cluster Survey (MICS) conducted in 2019 by UNICEF, a large-scale multidimensional household survey that is nationally representative. Standardized questionnaires are used to address child labor, violent discipline, parental involvement, education, child development, and socioeconomic and household characteristics [34].

### Sampling design and sample size

The MICS survey applies a two-stage cluster sampling technique to randomly select children between the ages of 5 and 17. The 2019 MICS is based on a sample of 40,617 mothers/caretakers who were surveyed, with 97.0% of them responding. On the basis of the expected sizes of the enumeration regions from the most recent population census, primary sampling units (PSUs) for the census were developed and chosen from each sample domain using systematic probability proportional to size (PPS) sampling processes. Among the eight administrative divisions (Dhaka, Mymensingh, Chittagong, Sylhet, Rajshahi, Rangpur, Barishal, and Khulna) of Bangladesh, MICS offers a thorough picture of children’s education. The main sampling strata were urban and rural areas within each district, and the sample of households was drawn in two stages [34]. A total of 31,196 children admitted to school last year were included in the study. Of these, 15,639 were enrolled in primary school, while those not enrolled were excluded. A total of 14,305 children aged 6–12 years were included, and the remaining children who were not aged 6–12 years were excluded [35]. The final sample of 14,278 children who started school at age 6 or older was included, whereas those who started school before 6 years were excluded because the official primary school entrance age in Bangladesh is 6 years [25]. Fig 1 shows a schematic diagram of the study sample.

**Fig 1:**
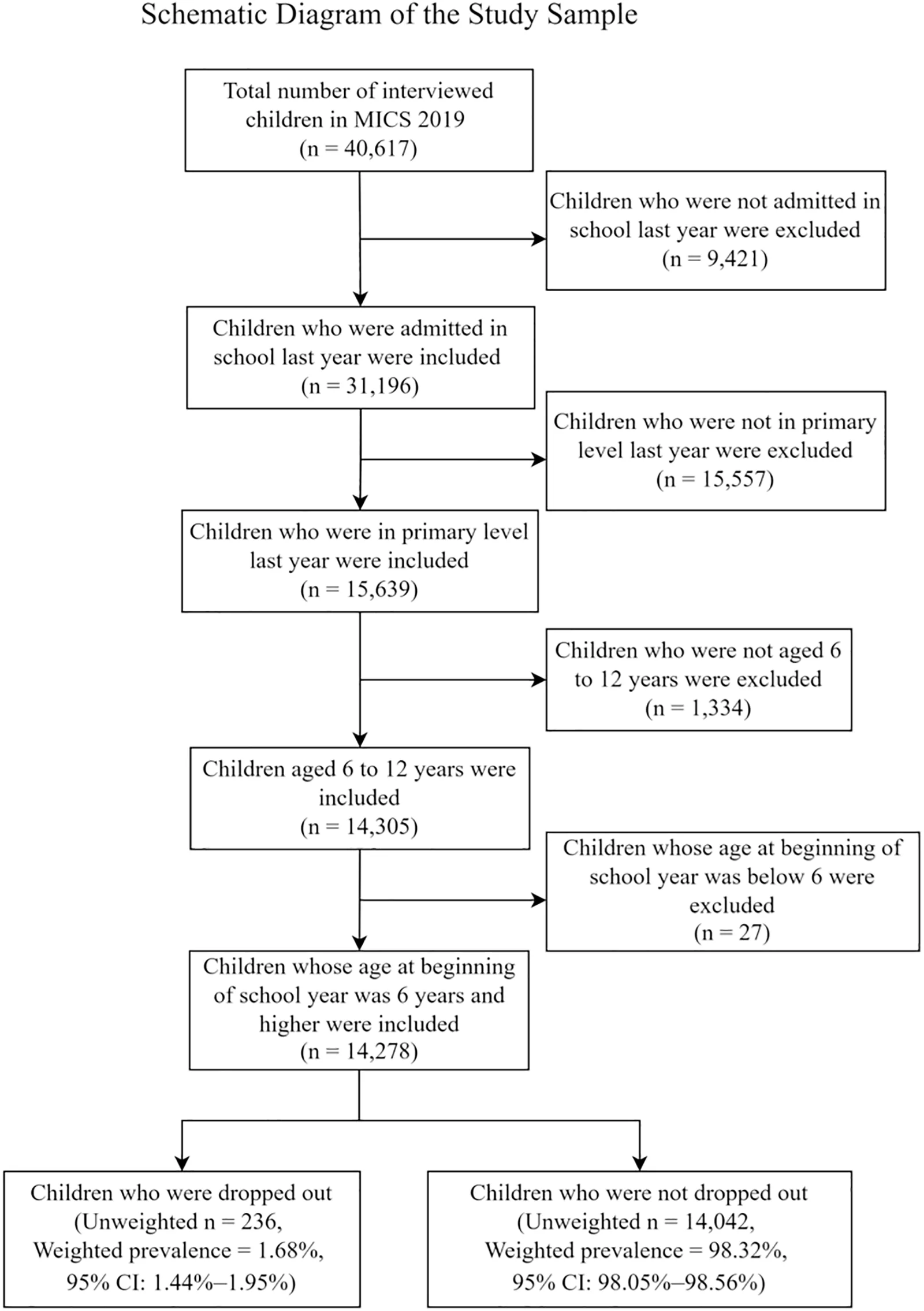
Schematic diagram of the study sample.

### Outcome variable

To create our outcome variable (dropout status), we assigned a value of 1 to each child on the basis of not attending school or an early childhood program during the current school year but was admitted last year, indicating a “yes” response; otherwise, 0 indicated “no”. Among the observed sample, the weighted prevalence of dropout was 1.68% (95% CI: 1.44%−1.95%, unweighted n = 236), while 98.32% remained in school (95% CI: 98.05%−98.56%, unweighted n = 14,042) (Fig 1).

### Risk factors

To determine the possible risk factors affecting dropout status, a set of factors was selected: child’s age (6–8 years and 9–12 years), sex, child’s age at the beginning of the school year (6–8 years and 9–12 years) [36], children’s functional difficulties, involvement in economic activities, needed to be physically punished to be brought up properly, severe physical punishment, mother’s education (preprimary or none, primary, secondary and higher secondary or above), mother’s functional difficulties and whether she had another child under 5, father’s education (preprimary or none, primary, secondary and higher secondary or above), area (urban or rural), division, gender of the household head, ethnicity of the household head (Bengali or others), religion (Islam, Hinduism, Christianity, Buddhism), household size (up to 4 or more than 4) [37], household wealth index [38], household had electricity, household had internet, types of toilet facility (improved or unimproved), salt iodization, sources of water (improved or unimproved), child had 3 or more books to read at home, child read books or was read to at home, and child spoke the same language at home and school.

Age at the beginning of the school year was obtained from the MICS variable *schage* and categorized into 6–8 years and 9–12 years. Child’s involvement in economic activities was categorized as “yes” if he/she worked or helped in the garden, helped in the family business, produced or sold articles, and engaged in other activities for income; otherwise “no”. A child faced severe physical discipline if he/she was hit or slapped on the face, head, or ears or beaten up as hard as possible; otherwise, “no”. The types of toilet facilities were categorized into improved (flush toilet, flush to piped sewer system, flush to septic tank, flush to pit latrine, pit latrine with slab and ventilated improved pit latrine) and unimproved (flash to open drain, pit latrine without slab, hanging toilet, bush/field) [39]. Salt iodization was categorized as “yes” if the iodine level was between 0 and 15 ppm or above and “no” if the iodine level was 0 ppm or no salt in the household [40]. Sources of water were categorized into improved (piped water, tubewell, rainwater, water kiosk) and unimproved (unprotected well, spring, river/pond) [41]. Children read books or were read to at home was measured using *FL6A* (whether the child reads books at home), and *FL6B* (whether someone reads books to the child at home). These two variables were combined and categorized as “Yes” when either activity was reported and “No” when neither activity was reported.

### Statistical analysis

We used the *svyset* (declare data as survey data) command in Stata to account for the complex survey design [42]. The primary sampling unit was cluster number (*HH1*), the sampling weight was children 5–17’s sample weight (*fsweight*), and *stratum* was specified as the stratification variable. The survey design was incorporated into the estimation of percentages, design-based tests of association, and regression analyses, including the calculation of p-values and 95% confidence intervals. The eligible study population was defined as a survey subpopulation using the *subpop* option so that the survey design information was retained while restricting estimation to the target population. Missing observations were retained in the dataset during descriptive and bivariate analyses. For the multivariable analysis, a complete-case approach was used. Bivariate analysis using two-way cross-tabulation with Design-based F tests (Rao-Scott adjusted chi-square test) was performed to evaluate the associations between dropout and each of the 26 variables. Seventeen variables with P-value ≤ 0.20 were retained for subsequent crude regression analyses, following a staged variable selection approach used in previous epidemiological studies [43]. We used logistic, robust Poisson, and log-binomial regression models to assess and evaluate the associations among dropout, learning environment at home, and associated risk factors. Logistic regression model is widely used for binary outcomes and provides odds ratios (ORs) that are useful for assessing risk factors [44]. The log-binomial regression model directly estimates prevalence or risk ratios (RRs), making it more suitable for cross-sectional studies since the risk ratio is simpler for nonspecialists to comprehend and explain than the odds ratio is [45]. On the other hand, robust Poisson regression model is an alternative approach that uses robust error variance to provide risk ratio estimates while addressing issues of overestimated errors in binary outcomes [46]. Crude models were employed for each of the mentioned models, and we included the risk factors in the adjusted models on the basis of an arbitrary p-value threshold of ≤ 0.20. A liberal screening threshold was used to avoid prematurely excluding potentially important predictors or confounding variables that may become significant after adjustment, consistent with established regression model-building recommendations [47–49]. Additionally, with a cutoff value of 5.00, we used the variance inflation factor (VIF) value to analyze multicollinearity in the final model [50]. All variables, except for the child’s age and age at the beginning of the school year, had VIF values less than 5.00. Therefore, the child’s age was dropped from the adjusted model to mitigate multicollinearity issues. Each of the adjusted models was then employed. All models converged successfully. The log-binomial and robust Poisson models estimated adjusted risk ratios (ARRs) with 95% confidence intervals, whereas logistic regression estimated adjusted odds ratios (AORs) with 95% confidence intervals. Following the complete-case approach, the final analytic sample for the multivariable analysis comprised 10,813 children. Two further sensitivity analyses were performed to test the robustness of the main findings. First, because missing observations were present for the key exposure variable “Child read books or are read to at home”, missing values were alternatively classified as “No” and “Yes,” and the survey-weighted log-binomial regression model was re-estimated under each assumption. Second, a restricted survey-weighted log-binomial regression model was fitted using a theory-based set of covariates comprising child’s sex, age at the beginning of the school year, functional difficulties, involvement in economic activities, severe physical discipline, maternal education, paternal education, household wealth, and home reading activity. The results of these sensitivity analyses were compared with those of the primary complete-case model. We used evaluation metrics such as log-likelihood, Akaike information criterion (AIC), and Bayesian information criterion (BIC) values to compare all the models to find the best model. The higher value of log-likelihood as well as the lowest values of AIC and BIC indicate a better fit of the data after accounting for model complexity (i.e., the number of model parameters) [51].

### Ethics statement

This study relied on secondary analysis of publicly available data from Bangladesh Multiple Indicator Cluster Survey (MICS). The Bangladesh Bureau of Statistics (BBS) and UNICEF have acquired ethical clearance for the original survey following national and international ethical standards. Informed consent was obtained from all participants or parents/guardians prior to data collection. The MICS dataset was de-identified and was made available to the researcher without further ethical approval.

## Results

From bivariate analysis using two-way cross-tabulation with Design-based F tests (Rao-Scott adjusted chi-square test), seventeen variables were included in the crude model at a p-value ≤ 0.20 threshold (child’s age, sex, age at the beginning of the school year, child’s functional difficulties, child faces severe physical discipline, involvement in economic activities, mother’s education, father’s education, division, sex of household head, household wealth index, household has internet, type of toilet facility, salt iodization, sources of water, child has 3 or more books at home, child reads books or are read to at home), as shown in Table 1.

**Table 1:** Sample characteristics of children by dropout status.

Characteristics	Dropout		P-Value
	No	Yes	
	Unweighted n (Weighted row %)	Unweighted n (Weighted row %)	
**Total**	14,042 (98.32)	236 (1.68)
***Child Characteristics***			
**Child’s sex**			
Male	6,904 (97.46)	168 (2.54)	<0.001
Female	7,138 (99.15)	68 (0.85)	
**Child’s age**			
6 to 8 years	4,692 (98.97)	46 (1.03)	0.001
9 to 12 years	9,350 (98.03)	190 (1.97)	
**Age at the beginning of the school year**			
6 to 8 years	5,092 (98.89)	52 (1.11)	0.002
9 to 12 years	8,950 (98.03)	184 (1.97)	
**Child had functional difficulties**			
Yes	993 (97.00)	27 (3.00)	0.006
No	12,994 (98.44)	207 (1.56)	
**Child’s involvement in economic activities**			
Yes	1,345 (97.00)	42 (3.00)	<0.001
No	12,672 (98.46)	194 (1.54)	
***Child Discipline***			
**Child needed to be physically punished to be brought up properly**			
Yes	3,473 (98.24)	67 (1.76)	0.588
No	6,521 (98.40)	106 (1.60)	
**Child experienced severe physical discipline**			
Yes	4,235 (98.05)	70 (1.95)	0.181
No	9,805 (98.45)	165 (1.55)	
***Parental Characteristics***			
**Mother’s education**			
Pre-primary or none	2,986 (97.84)	75 (2.16)	0.009
Primary	3,868 (97.92)	86 (2.08)	
Secondary	5,993 (98.74)	67 (1.26)	
Higher secondary or above	1,195 (99.09)	8 (0.91)	
**Mother had functional difficulties**			
Yes	359 (97. 92)	8 (2. 08)	0.557
No	12,612 (98. 36)	199 (1. 64)	
**Mother had another child under 5**			
Yes	5,269 (98.33)	82 (1.67)	0.974
No	8,773 (98.32)	154 (1.68)	
**Father’s education**			
Pre-primary or none	3,457 (97.80)	78 (2.20)	0.071
Primary	3,503 (98.00)	72 (2.00)	
Secondary	3,324 (98.73)	39 (1.27)	
Higher secondary or above	1,513 (98.64)	16 (1.36)	
***Household Characteristics***			
**Area**			
Urban	2,620 (98.48)	38 (1.52)	0.546
Rural	11,422 (98.28)	198 (1.72)	
**Division**			
Barishal	1,365 (98.48)	22 (1.52)	0.006
Chattogram	2,542 (98.30)	38 (1.70)	
Dhaka	2,764 (98.63)	41 (1.37)	
Khulna	2,242 (98.54)	33 (1.46)	
Mymensingh	734 (96.38)	28 (3.62)	
Rajshahi	1,706 (98.41)	31 (1.59)	
Rangpur	1,750 (98.69)	21 (1.31)	
Sylhet	939 (98.14)	22 (1.86)	
**Household head’s sex**			
Male	12,577(98.21)	222(1.79)	0.004
Female	1,465(99.24)	14(0.76)	
**Ethnicity of household head**			
Bengali	13,704 (98.31)	232 (1.69)	0.247
Other	338 (98.07)	4 (0.93)	
**Religion**			
Islam	12,458 (98.22)	226 (1.78)	0.252
Hinduism	1,240 (99.43)	7 (0.57)	
Christianity	71 (100.00)	0 (0.00)	
Buddhism	273 (99.02)	3 (0.98)	
**Household size**			
Up to 4	7,083 (98.39)	109 (1.61)	0.677
More than 4	6,959 (98.28)	127 (1.72)	
**Household wealth index**			
Poor	3,428 (97.68)	78 (2.32)	0.013
Middle	5,994 (98.35)	100 (1.65)	
Rich	4,565 (98.71)	56 (1.29)	
**Households had electricity**			
Yes	12,635 (98.31)	214 (1.69)	0.649
No	1,407 (98.48)	22 (1.52)	
**Household had internet**			
Yes	4,551 (98.77)	54 (1.23)	0.016
No	9,488 (98.09)	182 (1.91)	
**Type of toilet facility**			
Improved	11,662 (98.52)	172 (1.48)	0.001
Unimproved	2,380 (97.41)	64 (2.59)	
**Salt iodization**			
Yes	10,206 (98.44)	161 (1.56)	0.134
No	3,835 (98.01)	75 (1.99)	
**Sources of water**			
Improved	13,729 (98.34)	229 (1.66)	0.196
Unimproved	313 (97.02)	7 (2.98)	
***Learning Environment at home***			
**Child had 3 or more books to read at home**			
Yes	470 (99.25)	3 (0.75)	0.197
No	13,098 (98.26)	229 (1.74)	
**Child read books or are read to at home**			
Yes	12,350 (98.93)	127 (1.07)	<0.001
No	360 (84.91)	58 (15.09)	
**Child spoke same language at home and school**			
Yes	13,824 (98.33)	232 (1.67)	0.488
No	218 (97.58)	4 (2.42)	

Table 1 shows that among the 14,278 children, 236 (1.68%) were primary school dropouts and 14,042 (98.32%) remained in school. Dropout proportion was more common among male children than female children (2.54% vs. 0.85%) and among children aged 9–12 years than those aged 6–8 years (1.97% vs. 1.03%). Similarly, dropout was higher among children aged 9–12 years at the beginning of the school year than among those aged 6–8 years (1.97% vs. 1.11%). Children with functional difficulties had a higher dropout proportion than those without such difficulties (3.00% vs. 1.56%). Dropout was also more common among children involved in economic activities than among those who were not (3.00% vs. 1.54%). The proportion of dropout was 1.95% among children who experienced severe physical discipline and 1.55% among those who did not.

In addition, dropout proportions differed across categories of parental education. The dropout proportion was 2.16% among children whose mothers had pre-primary education or no education, compared with 0.91% among those whose mothers had higher secondary education or above. Similarly, the dropout proportion was 2.20% among children whose fathers had pre-primary education or no education, compared with 1.36% among those whose fathers had higher secondary education or above. Geographically, the highest dropout proportion was observed in Mymensingh (3.62%), while the lowest was observed in Rangpur (1.31%), followed by the other divisions, Sylhet (1.86%), Rajshahi (1.59%), Barishal (1.52%), Chattogram (1.70%), Dhaka (1.37%), and Khulna (1.46%).

The dropout proportion was 0.76% among the children whose household head was female than among those whose household head was male (1.79%). In terms of household facilities, the dropout rates were 1.23% for children with internet access and 1.91% for children without internet access. The dropout proportions were 1.48% for children in households with improved toilet facilities and 2.59% for those with unimproved facilities. The dropout proportions were 1.56% among children who lived in households with iodized salt and 1.99% among those who lived without iodized salt. For water sources, the corresponding dropout proportions were 1.66% for improved sources and 2.98% for unimproved sources. Children who read books or who were read at home had a lower dropout rate (1.07%) than those who did not (15.09%). Similarly, the dropout proportion was 0.75% among children who had three or more books at home, compared with 1.74% among those who had fewer than three books. Missing observations were present for several study variables, particularly whether the child needed to be physically punished to be brought up properly (28.79%), father’s education (15.94%), and child read books or are read to at home (9.69%). The number and proportion of missing observations for all study variables are presented in S1 Table.

S2 Table displays the evaluation metrics of the log-binomial, logistic, and robust Poisson regression models. For log-binomial regression model, the AIC and BIC estimates (AIC = 1439.431, BIC = 1636.221) were lower than the logistic regression model (AIC = 1440.337, BIC = 1637.127) and robust Poisson regression model (AIC = 1453.771, BIC = 1650.560). Moreover, the log-binomial regression model had a higher log-likelihood of −692.716, which was better fitted than logistic regression (log-likelihood = −693.169) and robust Poisson regression (log-likelihood = −699.885) models. The log-binomial regression model, which had lower AIC and BIC values and a higher log-likelihood, was found to be the best fit for this study.

Table 2 presents the findings of the crude model and the adjusted model exploring the relationship between the outcome and each risk factor using the log-binomial regression model. From the crude model, fifteen variables were included in the adjusted model at p-value≤ 0.20 and VIFs < 5.00 (sex, age at the beginning of school year, child’s functional difficulties, child faces severe physical discipline, involvement in economic activities, mother’s education, father’s education, division, sex of household head, household wealth index, household has internet, type of toilet facility, salt iodization, sources of water, child reads books or are read to at home). The results of the multivariable associations for the logistic and robust Poisson regression models are provided in S3 Table.

**Table 2:** Factors associated with the dropout status of children.

Characteristics	Log-Binomial Regression			
	Crude RR (95% CI)	P-value	Adjusted RR (95% CI)	P-value
**Child’s sex**				
Female	Reference	–	Reference	–
Male	2.30 (2.15, 4.18)	<0.001	2.44 (1.63, 3.64)	<0.001
**Child’s age**				
6 to 8 years	Reference	–		
9 to 12 years	1.92 (1.30, 2.82)	0.001		
**Age at beginning of school year**				
6 to 8 years	Reference	–	Reference	–
9 to 12 years	1.78 (1.23, 2.58)	0.002	1.83 (1.15, 2.93)	0.012
**Child had functional difficulties**				
No	Reference	–	Reference	–
Yes	1.92 (1.20, 3.05)	0.006	1.41 (0.78, 2.53)	0.254
**Child’s involvement in economic activities**				
No	Reference	–	Reference	–
Yes	1.94 (1.33, 2.84)	0.001	1.57 (0.97, 2.53)	0.066
**Child experienced severe physical discipline**				
No	Reference	–	Reference	–
Yes	1.26 (0.90, 1.75)	0.182	1.19 (0.81, 1.76)	0.382
**Mother’s education**				
Higher secondary or above	Reference	–	Reference	–
Secondary	1.39 (0.57, 3.38)	0.471	0.91 (0.31, 2.64)	0.859
Primary	2.29 (0.96, 5.49)	0.062	0.92 (0.30, 2.82)	0.886
Pre-primary or none	2.38 (0.99, 5.73)	0.053	0.95 (0.30, 3.02)	0.933
**Father’s education**				
Higher secondary or above	Reference	–	Reference	–
Secondary	0.94 (0.47, 1.87)	0.855	1.15 (0.51, 2.57)	0.736
Primary	1.47 (0.77, 2.79)	0.241	1.35 (0.58, 3.10)	0.485
Pre-primary or none	1.62 (0.86, 3.05)	0.137	1.27 (0.56, 2.88)	0.572
**Division**				
Barishal	Reference	–	Reference	–
Chattogram	1.12 (0.60, 2.08)	0.721	2.26 (0.96, 5.36)	0.063
Dhaka	0.90 (0.50, 1.64)	0.733	1.31 (0.59, 2.91)	0.514
Khulna	0.96 (0.50, 1.87)	0.912	1.66 (0.67, 4.14)	0.274
Mymensingh	2.39 (1.25, 4.58)	0.009	3.82 (1.59, 9.18)	0.003
Rajshahi	1.05 (0.56, 1.97)	0.881	1.37 (0.57, 3.30)	0.483
Rangpur	0.86 (0.42, 1.76)	0.686	1.86 (0.76, 4.56)	0.172
Sylhet	1.23 (0.63, 2.40)	0.546	1.71 (0.69, 4.28)	0.249
**Household head’s sex**				
Female	Reference	–	Reference	–
Male	2.36 (1.30, 4.28)	0.005	3.54 (0.47, 26.46)	0.219
**Household wealth index**				
Poor	Reference		Reference	–
Middle	0.71 (0.50, 1.02)	0.065	1.21 (0.75, 1.97)	0.433
Rich	0.56 (0.37, 0.83)	0.004	1.78 (0.97, 3.28)	0.063
**Household had internet**				
Yes	Reference	–	Reference	–
No	1.55 (1.08, 2.21)	0.017	1.37 (0.80, 2.33)	0.250
**Type of toilet facility**				
Improved	Reference	–	Reference	–
Unimproved	1.75 (1.26, 2.45)	0.001	1.40 (0.92, 2.14)	0.120
**Salt iodization**				
Yes	Reference	–	Reference	–
No	1.27 (0.93, 1.75)	0.135	1.08 (0.70, 1.67)	0.731
**Sources of water**				
Improved	Reference	–	Reference	–
Unimproved	1.80 (0.73, 4.41)	0.199	1.53 (0.53, 4.39)	0.431
**Child had 3 or more books to read at home**				
Yes	Reference	–		
No	2.31 (0.62, 8.61)	0.212		
**Child read books or are read to at home**				
Yes	Reference	–	Reference	–
No	14.16 (9.94, 20.17)	<0.001	14.47 (9.30, 22.51)	<0.001

The results of the log-binomial regression adjusted model showed that the risk of dropping out for males was significantly increased by 2.44 times relative to that for females (ARR = 2.44, 95% CI: 1.63–3.64). Those between 9 and 12 years of age at the beginning of school year had a 1.83 times greater risk of dropping out than those aged 6–8 years (ARR = 1.83, 95% CI: 1.15–2.93). Compared with children from the Barishal division, those from Mymensingh were 3.82 times more likely to drop out (ARR = 3.82, 95% CI: 1.59–9.18). Children who neither read books nor were read to at home were 14.47 times more likely (ARR = 14.47, 95% CI: 9.30–22.51) to drop out of school than children who either read books or were read to at home (Table 2). Sensitivity analyses showed that when missing values for this variable were alternatively classified as “No” or “Yes,” the corresponding ARRs were 6.17 (95% CI: 4.38, 8.69, P-value < 0.001) and 11.02 (95% CI: 7.26, 16.72, P-value < 0.001), respectively (S4 Table). In the sensitivity analysis using the restricted covariate set, the association between child read books or was read to at home and dropout remained strong (ARR = 13.13, 95% CI: 8.76, 19.68, P-value < 0.001) (S5 Table). Overall, the sensitivity analyses confirmed that the association between limited home reading activity and school dropout was robust to different assumptions about missing exposure values and to the use of a restricted, theory-based covariate set. Although the magnitude of the association varied across analyses, its direction and statistical significance remained consistent.

## Discussion

In this study, we used 2019 MICS data to examine the associations between primary school dropout and child characteristics, household factors and the learning environment at home in Bangladesh. The analysis suggested that there was a statistically significant association between primary school dropout and the gender of children. Children’s age at the beginning of school year was also significantly associated. Additionally, divisional disparities and whether the child read books or was read to at home were significantly associated with dropout.

The dropout rate of primary school children was less than 2% in our research, where the dropout rate was higher in sub-Saharan Africa, and South and West Asia [52]. According to the UNICEF Regional Office for South Asia, an estimated 27 million children aged 5–13 years were out of school in Bangladesh, India, Pakistan, and Sri Lanka, including approximately 17 million primary school-aged children, highlighting the magnitude of educational exclusion in the region [53]. Moreover, about 10% of students drop out of school in the United Arab Republic (U.A.R.), China, Cambodia and Japan [54]. Thus, although Bangladesh has made considerable progress, identifying the factors that place certain children at greater risk of leaving school is essential for designing effective interventions.

In the child characteristics section, our research revealed that male children had a higher primary school dropout rate than female children. The results coincided with research conducted in Cambodia, China [55]. A previous study also revealed that boys tend to drop out of primary school more than girls do [16]. Boys are also at risk of dropping out due to gender norms that direct them into the economy and home obligations at an early age, while disengagement from school and poor school experiences may further increase their risk [56]. We also found that older children were at significantly higher risk of dropping out of school compared to their younger peers. This is in line with evidence from Bangladesh and Malawi that older children were more likely to drop out of education [29,57]. Older children may have more economic and household responsibilities including child labor and domestic work which may impact their education. In addition, being older than expected for their grade may be associated with higher risk of school dropout.

In the household characteristics section, our research revealed divisional disparities in dropout rates among primary school children. This finding aligns with a previous study in Bangladesh, which reported that dropout rates vary according to division [27]. Recent spatial analysis of primary school enrolment revealed substantial variation in enrolment rates across the region, particularly high enrolment rates in the western divisions and low enrolment rates in parts of eastern Bangladesh [58]. Finally, regarding the learning environment at home, our findings showed that children who neither read books nor were read to at home had a higher possibility of dropping out than children who either read books themselves or were read to at home. Previous research has shown that the home learning environment is associated with children’s literacy and school-readiness skills [59]. Longitudinal evidence has also shown that early shared reading is associated with later academic achievement through children’s language and early academic skills [60]. On the contrary, evidence from Côte d’Ivoire found that limited home literacy resources were associated with an increased risk of primary school dropout [61]. The overall results highlight the need to adopt context-specific strategies to encourage older children at the beginning of the school year, encourage home reading, ensure that boys stay in school, and tackle regional differences to decrease primary school dropout in Bangladesh.

## Strengths and limitations

There are several strengths in this study. The study utilized recent 2019 MICS datasets, which are nationally representative and collected using rigid sampling techniques. Notably, no current research has investigated the relationship between primary school dropout and household learning environment variables. Additionally, we employed various statistical models, including logistic, robust Poisson, and log-binomial models, to assess the associations between primary school dropout and risk factors. Furthermore, the log-binomial regression model enabled us to provide more interpretable results.

However, there are certain limitations to our study. The cross-sectional design of MICS data provides information for a single year. Time series data could be beneficial for more thorough investigations if multiple surveys from Bangladesh are considered. Merging multi-country survey data would help identify a wider array of risk factors and their impact on primary school dropouts. Combining data from secondary and upper secondary education levels could provide a better understanding of quitting trends and causes. As the data was secondary, we had no control over their quality, or the variables included. School-related factors potentially influencing dropouts, such as teacher and teaching quality and school infrastructure, were not available. In particular, reverse causality is possible for home reading activity, as children may have reduced reading activity after dropping out of school. Therefore, the strong association observed between home reading activity and dropout should not be interpreted as causal. Future research could address these limitations.

## Recommendations

From the findings of this study, it is recommended that governments, non-governmental organizations (NGOs), communities and families should prioritize interventions that support older children, promote home reading activities, and increase parental participation in children’s learning. Targeted strategies should also address the barriers that place boys at greater risk of dropping out and reduce regional disparities in educational access and school retention. Building reading culture at home and introducing educational support programs in the region may also increase opportunities for children to engage in learning and improve primary school retention. Based on the key factors found in this study stakeholders can jointly develop supportive learning environment to reduce primary school dropouts in Bangladesh.

## Conclusion

We conducted a thorough study based on the 2019 MICS datasets from Bangladesh to investigate the primary school dropouts and the associated risk factors. Our research indicated that children who neither read books nor were read to at home were more prone to primary school dropout. This study also illustrated geographical variations in children’s primary school dropouts. Gender and older age were also associated with primary school dropouts. These findings may inform future interventions to improve educational outcomes. By implementing targeted interventions, such as promoting reading activities at home, we can create a more equitable educational environment for all children. These findings will aid initiatives aimed at diminishing gender inequality, supporting older children, reducing regional disparities, and enhancing the home learning environment to reduce dropouts among primary school children.

## Supporting information

S1 Table Number and proportion of missing observations for study variables. (DOCX)

S2 Table Evaluation metrics of the models. (DOCX)

S3 Table Factors associated with the dropout status of children based on logistic regression and robust Poisson regression models. (DOCX)

S4 Table Sensitivity analysis under alternative assumptions for missing values of whether the child read books or was read to at home. (DOCX)

S5 Table Sensitivity analysis using a restricted covariate set. (DOCX)
