# Supplementary material for: Learning environment at home and primary school dropout status in Bangladesh: An analysis of multiple indicator cluster survey
Source: PLoS One. 2026 Oct 21;21(10):e0360931. doi: 10.1371/journal.pone.0360931 (PMC13686498; doi:10.1371/journal.pone.0360931)
Supplement: Supplementary file 1 [file pone.0360931.s001.docx]

| **Variable** | **Total n** | **Missing n** | **Missing %** |
| --- | --- | --- | --- |
| Dropout | 14278 | 0 | 0 |
| Child’s sex | 14278 | 0 | 0 |
| Child’s age | 14278 | 0 | 0 |
| Age at the beginning of the school year | 14278 | 0 | 0 |
| Child had functional difficulties | 14278 | 57 | 0.4 |
| Child’s involvement in economic activities | 14278 | 25 | 0.18 |
| Child needed to be physically punished to be brought up properly | 14278 | 4111 | 28.79 |
| Child experienced severe physical discipline | 14278 | 3 | 0.02 |
| Mother's education | 14278 | 0 | 0 |
| Mother had functional difficulties | 14278 | 0 | 0 |
| Mother had another child under 5 | 14278 | 0 | 0 |
| Father’s education | 14278 | 2276 | 15.94 |
| Area | 14278 | 0 | 0 |
| Division | 14278 | 0 | 0 |
| Household head’s sex | 14278 | 0 | 0 |
| Ethnicity of household head | 14278 | 0 | 0 |
| Religion | 14278 | 0 | 0 |
| Household size | 14278 | 0 | 0 |
| Household wealth index | 14278 | 57 | 0.4 |
| Households had electricity | 14278 | 0 | 0 |
| Household had internet | 14278 | 3 | 0.02 |
| Type of toilet facility | 14278 | 0 | 0 |
| Salt iodization | 14278 | 1 | 0.01 |
| Sources of water | 14278 | 0 | 0 |
| Child had 3 or more books to read at home | 14278 | 478 | 3.35 |
| Child read books or are read to at home | 14278 | 1383 | 9.69 |
| Child spoke same language at home and school | 14278 | 0 | 0 |
