# Supplementary material for: Learning environment at home and primary school dropout status in Bangladesh: An analysis of multiple indicator cluster survey
Source: PLoS One. 2026 Oct 21;21(10):e0360931. doi: 10.1371/journal.pone.0360931 (PMC13686498; doi:10.1371/journal.pone.0360931)
Supplement: Supplementary file 2 [file pone.0360931.s002.docx]

| **Model** | **Log-Likelihood** | **AIC** | **BIC** |
| --- | --- | --- | --- |
| Log-Binomial Regression | -692.716 | 1439.431 | 1636.221 |
| Logistic Regression | -693.169 | 1440.337 | 1637.127 |
| Robust Poisson Regression | -699.885 | 1453.771 | 1650.560 |
