# Supplementary material for: Learning environment at home and primary school dropout status in Bangladesh: An analysis of multiple indicator cluster survey
Source: PLoS One. 2026 Oct 21;21(10):e0360931. doi: 10.1371/journal.pone.0360931 (PMC13686498; doi:10.1371/journal.pone.0360931)
Supplement: Supplementary file 3 [file pone.0360931.s003.docx]

| **Characteristics** | **Logistic Regression** | | **Robust Poisson Regression** | |
| --- | --- | --- | --- | --- |
|  | **Adjusted**  **OR (95% CI)** | **P-value** | **Adjusted**  **RR (95% CI)** | **P-value** |
| **Child’s sex** |  |  |  |  |
| Female | Reference | - | Reference | - |
| Male | 2.69 (1.78, 4.06) | <0.001 | 2.54 (1.74, 3.71) | <0.001 |
| **Age at beginning of school year** | | | | |
| 6-8 years | Reference | - | Reference | - |
| 9-12 years | 1.87 (1.13, 3.09) | 0.015 | 1.77 (1.12, 2.81) | 0.014 |
| **Child had functional difficulties** | | | | |
| No | Reference | - | Reference | - |
| Yes | 1.43 (0.72, 2.87) | 0.310 | 1.40 (0.75, 2.62) | 0.290 |
| **Child's involvement in economic activities** | | | | |
| No | Reference | - | Reference | - |
| Yes | 1.37 (0.85, 2.19) | 0.192 | 1.36 (0.89, 2.07) | 0.155 |
| **Child experienced severe physical discipline** | | | | |
| No | Reference | - | Reference | - |
| Yes | 1.29 (0.85, 1.95) | 0.228 | 1.25 (0.86, 1.82) | 0.245 |
| **Mother’s education** |  |  |  |  |
| Higher secondary or above | Reference | - | Reference | - |
| Secondary | 0.96 (0.34, 2.70) | 0.932 | 0.97 (0.37, 2.59) | 0.956 |
| Primary | 1.00 (0.34, 2.95) | 0.998 | 1.00 (0.36, 2.78) | 0.993 |
| Pre-primary or none | 0.99 (0.32, 3.03) | 0.986 | 1.00 (0.35, 2.86) | 0.996 |
| **Father’s education** |  |  |  |  |
| Higher secondary or above | Reference | - | Reference | - |
| Secondary | 1.10 (0.46, 2.64) | 0.837 | 1.08 (0.47, 2.48) | 0.856 |
| Primary | 1.47 (0.58, 3.72) | 0.411 | 1.43 (0.60, 3.45) | 0.422 |
| Pre-primary or none | 1.29 (0.52, 3.21) | 0.579 | 1.27 (0.54, 3.00) | 0.583 |
| **Division** |  |  |  |  |
| Barishal | Reference | - | Reference | - |
| Chattogram | 2.33 (0.92, 5.87) | 0.073 | 2.16 (0.93, 5.03) | 0.075 |
| Dhaka | 1.36 (0.57, 3.27) | 0.490 | 1.34 (0.59, 3.03) | 0.486 |
| Khulna | 1.73 (0.65, 4.58) | 0.271 | 1.61 (0.65, 4.01) | 0.303 |
| Mymensingh | 5.10 (1.91, 13.57) | 0.001 | 4.29 (1.78, 10.34) | 0.001 |
| Rajshahi | 1.46 (0.56, 3.82) | 0.444 | 1.39 (0.57, 3.37) | 0.469 |
| Rangpur | 1.95 (0.75, 5.12) | 0.173 | 1.84 (0.76, 4.48) | 0.180 |
| Sylhet | 1.80 (0.66, 4.87) | 0.250 | 1.73 (0.67, 4.46) | 0.254 |
| **Household head’s sex** |  |  |  |  |
| Female | Reference | - | Reference | - |
| Male | 3.61 (0.43, 30.53) | 0.239 | 3.31 (0.43, 25.51) | 0.251 |
| **Household wealth index** | | | | |
| Poor | Reference | - | Reference | - |
| Middle | 1.05 (0.64, 1.74) | 0.834 | 1.07 (0.68, 1.69) | 0.759 |
| Rich | 1.67 (0.88, 3.19) | 0.117 | 1.63 (0.90, 2.95) | 0.105 |
| **Household had internet** |  |  |  |  |
| Yes | Reference | - | Reference | - |
| No | 1.35 (0.76, 2.39) | 0.299 | 1.34 (0.79, 2.27) | 0.283 |
| **Type of toilet facility** |  |  |  |  |
| Improved | Reference | - | Reference | - |
| Unimproved | 1.45 (0.93, 2.27) | 0.104 | 1.40 (0.93, 2.09) | 0.105 |
| **Salt iodization** |  |  |  |  |
| Yes | Reference | - | Reference | - |
| No | 1.17 (0.75, 1.81) | 0.485 | 1.15 (0.77, 1.71) | 0.502 |
| **Sources of water** |  |  |  |  |
| Improved | Reference | - | Reference | - |
| Unimproved | 1.67 (0.48, 5.85) | 0.421 | 1.63 (0.52, 5.17) | 0.404 |
| **Child read books or were read to at home** | | | |  |
| Yes | Reference | - | Reference | - |
| No | 18.17 (11.24, 29.36) | <0.001 | 13.84 (9.42, 20.34) | <0.001 |
