# Supplementary material for: Learning environment at home and primary school dropout status in Bangladesh: An analysis of multiple indicator cluster survey
Source: PLoS One. 2026 Oct 21;21(10):e0360931. doi: 10.1371/journal.pone.0360931 (PMC13686498; doi:10.1371/journal.pone.0360931)
Supplement: Supplementary file 4 [file pone.0360931.s004.docx]

| **Variables** | **Log-Binomial Regression** | | | |
| --- | --- | --- | --- | --- |
|  | **Analytical Sample: 10,813** | | **Analytical Sample: 10,817** | |
|  | **Primary ARR (95% CI)** | **Primary P-value** | **Sensitivity ARR (95% CI)** | **Sensitivity P-value** |
| **Child’s sex** | | | | |
| Female | Reference | - | Reference | - |
| Male | 2.44 (1.63, 3.64) | <0.001 | 2.41 (1.61, 3.63) | <0.001 |
| **Age at beginning of school year** | | | | |
| 6 to 8 years | Reference | - | Reference | - |
| 9 to 12 years | 1.83 (1.15, 2.93) | 0.012 | 1.90 (1.21, 2.98) | 0.005 |
| **Child had functional difficulties** | | | | |
| No | Reference | - | Reference | - |
| Yes | 1.41 (0.78, 2.53) | 0.254 | 1.41 (0.79, 2.53) | 0.247 |
| **Child's involvement in economic activities** | | | | |
| No | Reference | - | Reference | - |
| Yes | 1.57 (0.97, 2.53) | 0.066 | 1.41 (0.88, 2.24) | 0.149 |
| **Child experienced severe physical discipline** | | | | |
| No | Reference | - | Reference | - |
| Yes | 1.19 (0.81, 1.76) | 0.382 | 1.31 (0.90, 1.92) | 0.164 |
| **Mother’s education** | | | | |
| Higher secondary or above | Reference | - | Reference | - |
| Secondary | 0.91 (0.31, 2.64) | 0.859 | 0.95 (0.33, 2.74) | 0.925 |
| Primary | 0.92 (0.30, 2.82) | 0.886 | 1.00 (0.33, 3.03) | 0.996 |
| Pre-primary or none | 0.95 (0.30, 3.02) | 0.933 | 1.08 (0.35, 3.37) | 0.891 |
| **Father’s education** | | | | |
| Higher secondary or above | Reference | - | Reference | - |
| Secondary | 1.15 (0.51, 2.57) | 0.736 | 1.14 (0.51, 2.56) | 0.750 |
| Primary | 1.35 (0.58, 3.10) | 0.485 | 1.42 (0.61, 3.29) | 0.415 |
| Pre-primary or none | 1.27 (0.56, 2.88) | 0.572 | 1.40 (0.61, 3.22) | 0.429 |
| **Household wealth index** | | | | |
| Poor | Reference | - | Reference | - |
| Middle | 1.21 (0.75, 1.97) | 0.433 | 0.95 (0.61, 1.47) | 0.807 |
| Rich | 1.78 (0.97, 3.28) | 0.063 | 1.16 (0.69, 1.95) | 0.576 |
| **Child read books or are read to at home** | | | | |
| Yes | Reference | - | Reference | - |
| No | 14.47 (9.30, 22.51) | <0.001 | 13.13 (8.76, 19.68) | <0.001 |

**Note:** The primary model was additionally adjusted for household division, sex of household head, internet access, toilet facility, iodized salt, and water source. The sensitivity model was restricted to the variables presented in the table.
