# Supplementary material for: Learning environment at home and primary school dropout status in Bangladesh: An analysis of multiple indicator cluster survey
Source: PLoS One. 2026 Oct 21;21(10):e0360931. doi: 10.1371/journal.pone.0360931 (PMC13686498; doi:10.1371/journal.pone.0360931)
Supplement: Supplementary file 5 [file pone.0360931.s005.docx]

| **Different approaches of missing values handling** | **Analytical Sample** | **ARR (95% CI)** | **P-value** |
| --- | --- | --- | --- |
| Primary complete-case analysis | 10,813 | 14.47 (9.30, 22.51) | <0.001 |
| Missing values classified as “No” | 11,926 | 6.17 (4.38, 8.69) | <0.001 |
| Missing values classified as “Yes” | 11,926 | 11.02 (7.26, 16.72) | <0.001 |

**Variable: Children who did not read books at home**
